# Microscopic

**Published:** 1877-07

**Authors:** 


### EDITORIAL.

### MICROSCOPIC.

We have just received from Prof. H. S. Chase, a contribution of one hundred specimens of sections of teeth, nicely mounted for the Ohio College of Dental Surgery.

This Collection is a valuable one, for which Prof. Chase will accept the thanks of the Faculty of the Institution. Of preparations of this kind, this Institution now has available, a large and varied collection.

